# O.3.1-8 The western research hub for physical activity and health: answering the World Health Organization’s call to implement the global action plan on physical activity

**DOI:** 10.1093/eurpub/ckad133.145

**Published:** 2023-09-11

**Authors:** Jane Thornton, Reilly Kristen

**Affiliations:** Fowler Kennedy Sport Medicine Clinic, London, ON, Canada; Western Centre for Public Health and Family Medicine, Schulich School of Medicine and Dentistry, Western University, London, ON, Canada; Department of Epidemiology and Biostatistics, Schulich School of Medicine and Dentistry, London, ON, Canada; School of Kinesiology, Western University, London, ON, Canada; jsthornt@uwo.ca; Fowler Kennedy Sport Medicine Clinic, London, ON, Canada; Western Centre for Public Health and Family Medicine, Schulich School of Medicine and Dentistry, Western University, London, ON, Canada

## Abstract

**Purpose:**

Physical activity provides a multitude of health benefits yet only half of Canadians meet national physical activity guidelines for health. In response to the global epidemic of physical inactivity, the World Health Organization (WHO) created the Global Action Plan on Physical Activity (GAPPA) to help countries prioritize decreasing physical inactivity. In 2021, we formed the Western Research Hub for Physical Activity and Health (The Hub) to respond to this call to action from the WHO. Our purpose is to advocate for and implement meaningful, sustainable and culturally relevant physical activity opportunities within our community and beyond. Our team is comprised of 30 junior and senior university scholars and a Community Advisory Council of local organization representatives and patient partners who inform and co-design our work.

**Project description:**

Through ongoing discussions with our members, we learned that while the GAPPA provides a clear roadmap, funding for such implementation projects is scarce and researchers, community organizations, and policymakers feel they do not have the necessary expertise to undertake implementation or scale-up projects. To address these gaps, we created The Hub’s Implementation Support Service. This service provides those working on GAPPA-related projects with support from our team in the form of: implementation resources (frameworks and toolkits); advice from implementation scientists; regular meetings to troubleshoot implementation barriers; networking support to connect with relevant partners; and financial support (seed grants worth $5000 CAD). Funding applications are reviewed and selected by a committee of researchers, community organizations, and patient partners. The Implementation Support Service will undergo a process and impact evaluation by understanding perceptions of those delivering and receiving the implementation service, as well as determining the short- and long-term effects of the implementation projects we support.

**Conclusions:**

The Western Research Hub for Physical Activity and Health is the first and only collective of its kind aimed directly at addressing and implementing the WHO’s GAPPA framework by providing a centralized access point for implementation seed grants and services. By providing resources and facilitating access to physical activity, our Hub can support more individuals staying active for more of their lives, with implications for population-level health.

